# It's Not De Quervain Tenosynovitis – A Diagnosis to Consider in Persistent Wrist Pain

**DOI:** 10.31486/toj.21.0005

**Published:** 2021

**Authors:** Eric Schmidt, Yuka Kobayashi, Andrew W. Gottschalk

**Affiliations:** ^1^ Department of Internal Medicine, Oregon Health and Science University, Portland, OR; ^2^ Department of Family Medicine and Sports Medicine, Oregon Health and Science University, Portland, OR; ^3^ Department of Orthopedics, Sports Medicine Institute, Ochsner Clinic Foundation, New Orleans, LA

## CASE PRESENTATION

A 41-year-old, right-hand-dominant racquetball player presents after 8 months of chronic, intermittent left wrist pain requiring her to take time off work and sport. She recalls mild onset of diffuse left wrist pain following long racquetball practices that gradually increased in severity and later localized to the distal dorsal radial wrist. Previous treatments included brief cessation of racquetball and activity modifications at work, wrist bracing, elbow strap, occupational therapy, nonsteroidal anti-inflammatory drugs, and heat/ice. Magnetic resonance imaging (MRI) of her wrist demonstrated no abnormal findings. Bedside ultrasound showed fluid accumulation at the area of maximal tenderness along the proximal intersection of the first and second extensor compartment tendons. She asks, “How can I return to racquetball?”

## BACKGROUND

Intersection syndrome is a form of tenosynovitis involving the intersection of the musculotendinous junctions of the first and second extensor compartment tendons. The first compartment (abductor pollicis longus and extensor pollicis brevis) passes obliquely over the second compartment (extensor carpi radialis brevis and extensor carpi radialis longus). Repetitive extension-flexion of the wrist and friction at the crossover junction cause pain, swelling, and inflammation of the distal dorsal-radial forearm, classically proximal to the radial styloid.^1^

In the general population, the incidence of intersection syndrome ranges from 0.2% to 0.37%.^2-4^ The condition is associated with several sports, such as rowing, canoeing, racket sports, horseback riding, mountain biking, and skiing.^5,6^ Typically, patients present with forearm pain, swelling, and sometimes redness and crepitus 4 to 6 cm proximal to the radiocarpal joint (Figure). MRI shows tendon thickening and peritendinous edema acutely, but these changes may not be seen in chronic cases because of stenosing tenosynovitis.^3^

**Figure.**
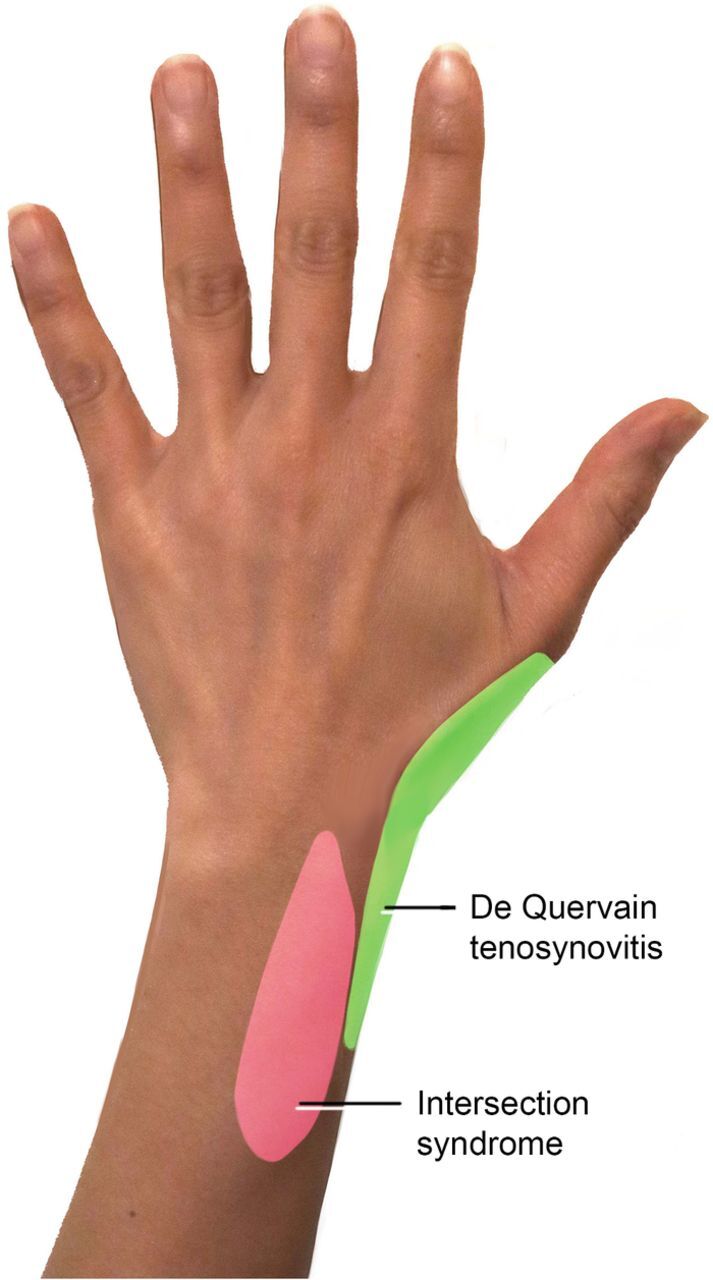
Regions of pain in De Quervain tenosynovitis and intersection syndrome.

## REVIEW OF EVIDENCE

Treatment of intersection syndrome involves 3 escalating levels, starting with conservative management and progressing to injections and surgical intervention.^2^ Approximately 60% of cases will resolve with conservative management in 2 to 3 weeks.^2,4,7-9^

In the first phase of conservative management, the patient incorporates rest, activity modification, and immobilization with a cock-up wrist or thumb spica splint in 15-degree extension.^2,8^ A short course of anti-inflammatory medications (eg, ibuprofen 600 mg 3 times daily or meloxicam 7.5 mg once daily for 7-14 days) is often necessary for pain control. Patients are also advised to use cryotherapy, elevation, compression, and taping across the dorsal forearm with force applied in the ulnar direction.^7^

Following the initial rest period of 2 to 3 weeks, the patient enters the second phase of conservative management that consists of gradual restoration of function and return to activity with progressive stretching, mobilization, and strength training for at least 4 to 6 weeks.^2^ Patients should follow the 10% rule to avoid relapse of symptoms: increase weight, repetitions, or distance by 10% per week.^8^

No compelling evidence-based rehabilitation protocols are available for intersection syndrome.^1^ If insufficient improvement is seen after phase 1 (the patient is unable to start returning to activities because of persistent symptoms) or phase 2 (the patient has a relapse of symptoms after returning to activities) of conservative management, injection therapies are offered.^2,8^

Several options for injection therapies are available to treat intersection syndrome. Corticosteroid injections are used most commonly, using a 1:1 mixture (approximately 0.5 to 1 mL) of corticosteroid (eg, Kenalog [triamcinolone acetonide] 40 mg/1 mL or 3 to 6 mg betamethasone sodium acetate) and anesthetic (eg, 1 mL lidocaine 1% without epinephrine) with a 1- to 1.5-inch, 22- to 25-gauge needle.^1^ Ultrasound-guided injections allow needle placement where the first dorsal compartment crosses over the second compartment.^1,8^ These injections provide resolution of symptoms within 10 days based on several case reports.^2,5,6,8^ The use of corticosteroid injections for de Quervain tenosynovitis (a condition similar to intersection syndrome) has level B evidence, but to our knowledge, no large series document intersection syndrome treatment outcomes in the literature.^10-12^ Overall risks of cortisone injections are minimal but include hypopigmentation, skin/subcutaneous fat atrophy or necrosis, tendon rupture, and infections.^13^

Prolotherapy is considered when the side effects of steroid therapy are a limiting factor and involves injecting a mixture of dextrose, sterile water, and lidocaine; although volumes vary, an example is 3 mL dextrose 50%, 5 mL saline 0.9%, and 2 mL lidocaine 2%.^1,14^ Prolotherapy is thought to induce a proinflammatory state that triggers the release of growth factors and ultimately collagen deposition, leading to strengthening of tissue.^1^ As with cortisone injections, evidence to support the use of prolotherapy in treating intersection syndrome is lacking.

Hydrodissection is the injection of high volumes of saline into the compartmental space to disrupt the neurovascular bundles that grow in the tendon mechanically and reduce adhesions.^15^ However, no studies have been published demonstrating the effectiveness of hydrodissection for intersection syndrome.

If injections fail to resolve symptoms of intersection syndrome, surgical intervention with tenosynovectomy, longitudinal incisions of the extensor retinaculum over the second compartment, is considered.^1^

## TAKEAWAY

Intersection syndrome is treated similarly to other types of tenosynovitis. However, more data are needed specifically regarding intersection syndrome to determine if clinical improvements are supported by the evidence.

## CASE RESOLUTION

After discussion of the proposed procedure, including risks and benefits, the patient elected ultrasound-guided corticosteroid injection with 0.5 mL Kenalog 40 mg/1 mL and 0.5 mL 1% lidocaine without epinephrine. She tolerated the injection well and restarted the home exercise program that she learned in occupational therapy. At 6-week follow-up, the patient reported substantial improvement in her pain and stated that she was able to return to racquetball with minimal symptoms.

## Notes

*This article meets the Accreditation Council for Graduate Medical Education and the American Board of Medical Specialties Maintenance of Certification competencies for Patient Care and Medical Knowledge.*
